# Engineering a skeletal muscle model to study extracellular vesicle dynamics

**DOI:** 10.1177/20417314261427541

**Published:** 2026-04-05

**Authors:** María Fernández-Rhodes, Rowan Rimington, Jacob Fleming, Andrew J. Capel, Owen G. Davies, Mark P. Lewis

**Affiliations:** 1 School of Sports Exercise and Health Sciences, Loughborough University, UK

**Keywords:** extracellular vesicles, skeletal muscle, myoblast, tissue engineering, model, 3D culture

## Abstract

Skeletal muscle (SM) functions both mechanically and as a secretory organ, releasing myokines and extracellular vesicles (EVs) involved in myogenic regulation and inter-tissue communication. While 3D bioengineered SM models are widely used for studying muscle physiology, few have been applied to investigate EV dynamics. This study optimised a 3D SM model to support mature myotube formation and evaluated its utility for SM-EV analysis. Myosin heavy chain expression was reduced at higher Matrigel^®^ concentrations (40%–60% v/v), highlighting the importance of matrix composition in model design. EVs were successfully isolated using size-exclusion chromatography and ultrafiltration, with yield influenced by cellular differentiation status. Common EV markers (Alix, CD9, CD63) were consistently expressed. Importantly, sarcoplasmic reticulum markers α- and β-sarcoglycan were identified in SM-EV preparations. These findings validate our SM model as a defined platform for studying SM-EV biology and defining molecular cargo.

## Introduction

Skeletal muscle (SM) accounts for 20%–50% of total body mass. It is a principal organ in energy expenditure, glucose metabolism and locomotion.^1,2^ 2D in vitro models have been widely applied in SM research, providing important advancements to our basic understanding of myogenesis.^3^ However, they often fail to recapitulate the complex organisation and functions of living tissues. The application of 3D bioengineered SM models provides an opportunity to better mimic the natural biodynamics of this tissue in a biologically and chemically defined environment.^4^ To date, SM models have been applied to deliver valuable insights into a wide variety of processes including SM pathophysiology and ageing.^5,6^

Over the last two decades, SM has become increasingly recognised as a secretory organ. Myoblasts and myotubes release cytokines and other peptides that are collectively termed “myokines” or “exerkines.” Myokines are responsible for the autocrine and paracrine coupling of SM with other tissues that include adipose tissue and bone.^7–9^ Many myokines and other molecules can be packaged in lipid delimited nanoparticles called extracellular vesicles (EVs) that are secreted/shed from the cell.^10–12^ These vesicles can be further subdivided into three groups: exosomes (size range ~30–200 nm, endosomal biogenesis), microvesicles (MVs; size range ~100–1000 nm) that bud from the external plasma membrane, and apoptotic bodies (ABs; size range ~0.5–2 µm). However, due to considerable overlap in their size ranges and protein composition, we shall collectively refer to them as EVs, as per published recommendations.^13^ SM-EVs are produced by both myoblasts and myotubes and express SM markers including myosin heavy chain (MyHC) and desmin.^10,14^ To date, SM-EVs have been associated with a range of processes that include myogenic regulation and tissue remodelling,^14–17^ inter-tissue and inter-organ communication^13,18–22^ and exercise adaptation.^12,23–25^ However, few studies have developed or utilised advanced 3D models to study SM-EV dynamics. Such models provide a defined environment for the study of fundamental processes prior to translation into highly complex in vivo systems.^26,27^

Bioengineered SM models are valuable in that they can provide controlled, scalable and reproduceable systems to study SM biology and function as a therapeutic testbed (e.g. drug screening).^28–31^ SM models have been broadly applied to study the effects of electrical or mechanical stimulation, as well as to model tissue regeneration following injury.^32–35^ They provide advantages over comparatively complex in vivo systems when attempting to study poorly defined processes such as intra- and inter-cellular communication events, which can become increasingly challenging in the presence of multiple cell types. This is particularly true for studies in which tracing the cellular origin(s) of EVs found in biological fluids such as blood plasma presents a major challenge due to the absence of defined tissue-specific markers. To further emphasise this challenge, one needs to appreciate that not only are EVs released into the circulation by practically every cell type within the body, but that EVs are also considerably outnumbered in the circulation (by a factor of 1 × 10^9^) by non-vesicular extracellular particles (NVEPs) such as lipoproteins with overlapping sizes and densities.^36,37^ To add further complexity, it has been estimated that only 1%–5% of circulating nanoparticles are likely to be of SM origin. This observation was made by quantifying the expression of α-sarcoglycan or miR-206, which represent prospective SM-EV markers.^38^ However, further research is required to confirm these findings. As such, the application of bioengineered models could provide an important transitory step in validating or identifying SM-EV biomarkers and in advancing our fundamental understanding of novel EV mediated processes that are essential to informing downstream in vivo studies.

## Methods

### 3D muscle constructs culture

**Figure 1.**
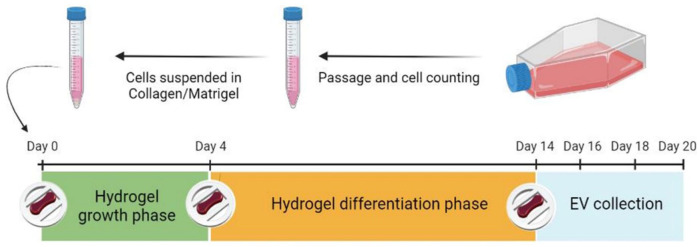
Diagram illustrating the fabrication of tissue engineered skeletal muscle C2C12 constructs. After passaging the cells, they were resuspended in the collagen/Matrigel^®^ mix. Constructs were incubated for 4 days in GM. From day 4 onwards, GM was replaced with myogenic DM. For EV collection, CM was recovered every 48 h intervals and SM-EVs isolated.

Collagen/Matrigel^®^ constructs were generated using C2C12 myoblasts in removable 50 μL moulds that were 3D printed via fused deposition modelling (FDM). SM constructs were formed by the addition of 65%, 45% or 25% v/v type I rat tail collagen, with 10% v/v of 10X minimal essential medium (MEM; *21430020, Gibco, UK*). This solution was subsequently neutralised by the addition of 5and 1 M sodium hydroxide (NaOH) dropwise, until a colour change to intense pink was observed. To the resulting matrix we added 20%, 40% or 60% v/v Corning^®^ Matrigel^®^ Matrix (*354234, Corning, Germany*).^28^ For subsequent EV studies, Matrigel^®^ comprised 20 % of the total gel volume. C2C12 murine myoblast cells were added at a seeding density of 4, 8, 12 and 16 × 10^6^ cells/mL per construct in a 5% v/v growth medium (GM; DMEM supplemented with 20% foetal bovine serum (FBS) and 1% penicillin/streptomycin (PS)) solution, before being transferred to pre-sterilised inserts and allowed to gel for 15–30 min at 37°C. Once optimised, 12 × 10^6^ cells/mL were applied per construct. For myogenic culture, GM was added for a period of 4 days, with daily media changes. At day 4, GM was replaced with differentiation medium (DM; DMEM supplemented with 2% EV-depleted horse serum (HS) and 1% of PS solution) for the remainder of the culture period.^28^ To deplete EVs the HS was centrifuged for 16 h at 120,000x*g*. For stimulation experiments, 5 or 20 mM of L-Leucine *(L8912, Sigma Aldrich, UK)* was added to the DM from day 6 with daily changes until day 14 (Figure 1).

### 3D skeletal muscle constructs sectioning

After performing functional studies, 3D SM constructs were fixed for at least 12 h in a 3.75% formaldehyde solution *(F8775, Sigma-Aldrich, Merck, KGaA, Darmstadt, Germany)*. This was followed by a dehydration process, using a 20% sucrose solution (w/v) in 1X Tris-buffered saline (TBS) for 24 h. The constructs were embedded in Tissue-Tek^®^ optimum cutting temperature (OCT) mounting medium *(25608-930, VWR, USA)* and frozen at −80°C with liquid. Once frozen 12 µm sections were prepared perpendicular to the longitudinal axis of the construct, using standard cryostat protocols. Three or more sections were placed in coated slides (*SuperFrost Plus*™ *Adhesion slides, FisherScientific, UK).*

### Immunocytochemistry (ICC)

Fixed slides of 12 µm diameter were permeabilised and exposed to a blocking solution (0.02% Triton X-100 and 0.5% goat serum in TBS 1X) for 1 h. After this step, engineered SM constructs were stained overnight with a solution of anti-myosin heavy chain (MyHC) antibody (1:500) and anti-F-Actin (1:500; *Novus Biologicals, Bio-Techne Ltd, UK*) with 0.005% goat serum in TBS 1X. After washing with TBS 1X, 3D constructs were incubated with a secondary antibody solution for MyHC using Alexa 488 goat anti-mouse (1:500; *Invitrogen, UK*), F-actin cytoskeleton (rhodamine phalloidin, 1:500; *15119325, FisherScientific, UK*) and nuclei (DAPI, 1:1,000; *10184322, FisherScientific, UK*) in TBS 1X for 1 h. Slides were then washed with dH_2_O and mounted using Fluoromount™ mounting medium *(F4680-25ML, Sigma-Aldrich, UK).*

Fluorescent images were captured using a Leica DM2500 microscope (*Leica, UK*). Morphological measures such as total nuclei per cross-sectional area (CSA), myotube density per 100 μm (number of myotubes measured intersecting a line drawn perpendicular to the long axes of the construct, average myotube width and averaged from five points per image) were all conducted manually. Total nuclei were calculated using an in-house macro for Image J 1.50a (*National Institutes of Health, USA*).^33^

### EV isolation: Ultrafiltration (UF) and size-exclusion chromatography (SEC)

Conditioned medium (CM) from engineered SM constructs was harvested every 48 h from day 14 onwards (Figure 1). An additional fraction was generated by combining equal volumes of fractions 14–20. CM was collected every 48 h, spun at 2000x*g* for 20 min to remove cellular debris and stored at −80°C for 24 h. CM was concentrated using Vivaspin^®^20 (100 kDa; *GE28-9323-63, Merck KGaA, Darmstadt, Germany*) ultrafiltration (UF) columns according to the manufacturer’s recommendations. Concentrated sample was submitted to a SEC column (*qEVoriginal/70 nm, IZON SCIENCE LTD, New Zealand*), with fractions 2–10 being collected and re-concentrated via UF, as per our optimised protocol.^39^

### Zetasizer particle measurements

Zetasizer Nano ZS (*Malvern Panalytical, UK*) was applied to provide zeta potential measurements for EV fractions. DTS1070 capillary cells (*Malvern Panalytical, UK*) were washed with isopropanol and ionised water and then dried before applying the sample. EVs were resuspended 1:20 in Dulbecco’s Phosphate Buffered Saline (DPBS) 1X and submitted to a capillary cell. Measurement time was 60 s at room temperature in monomodal mode using 50 mV, since DPBS 1X has high conductivity. Three repeats were measured per sample/condition to obtain a size measurement by dynamic light scattering (DLS) and zeta potential measurements.

### Bicinchoninic acid (BCA) protein assay

A Pierce™ BCA Protein Assay Kit (*23227, ThermoFisher Scientifics, UK*) was applied to quantify EV protein according to the supplier′s instructions. 25 µL of EV sample was loaded in a 96-well plate, followed by 200 µL BCA/copper complex solution. Absorbance was measured at 562 nm using a Thermo Scientific Varioskan Flash microplate reader *(ThermoFisher Scientifics, UK)* equipped with SkanIt Software 2.4.5 RE.

### Western blot

Sample was prepared at a concentration of 1 µg/mL in sample buffer 4X (SB4X; *1610747, BioRad, UK*) and lysis buffer (LB; 0.5% Triton X-100, EDTA 1X and protease inhibitors (*10085973, FisherScientific, UK*)). Each was boiled for 5 min at 98°C. Proteins present in the samples were separated in precast polyacrylamide gels (*4561083, BioRad, UK*), loading 5 μg protein and using three replicates per sample in all cases. Precision Plus Protein™ Dual Colour Standards were applied for estimation of molecular weight (*1610374, BioRad, UK*). Protein bands were transferred to Polyvinylidene fluoride (PVDF) membranes (*11544996, FisherScientific, UK*) that were blocked in EveryBlot blocking buffer (*12010020, BioRad, UK*) and washed in Tris buffer solution with 0.1% Tween20 (TBST; *Merck*™ *655204-100ML, FisherScientifics, UK*). Membranes were incubated with primary antibodies (Supplemental Table 1) overnight at 4°C with light agitation. The following day, membranes were washed three times and incubated with the appropriate secondary antibody (Supplemental Table 1) for 1 h at room temperature (RT). Protein bands were detected through chemiluminescence imaging using the ChemiDoc XRS+ system 3.2 (*1708265, BioRad, UK*) and Image Lab software 1.46 (*Life Science Research, BioRad, UK*). Image J 1.50a (*National Institutes of Health, USA*) was applied for WB band quantification.

### Statistical analysis

Graphs were generated using Origin Lab 2020 9.7.0.188 (*OriginLab Corporation, USA*) or GraphPad Prism 6 (GraphPad Software, San Diego, USA), with values presented as mean ± standard deviations (SDs). A 95% confidence interval (CI) was used for all functions. Pearson’s correlation-r was used to map correlations within samples. Analysis of variance (ANOVA) with Bonferroni post-hoc were performed using GraphPad Prism 6 (*GraphPad Software, San Diego, USA*). Differences were considered statistically significant at *p < 0.05 or **p < 0.01.

## Results

### 3D model composition

**Figure 2.**
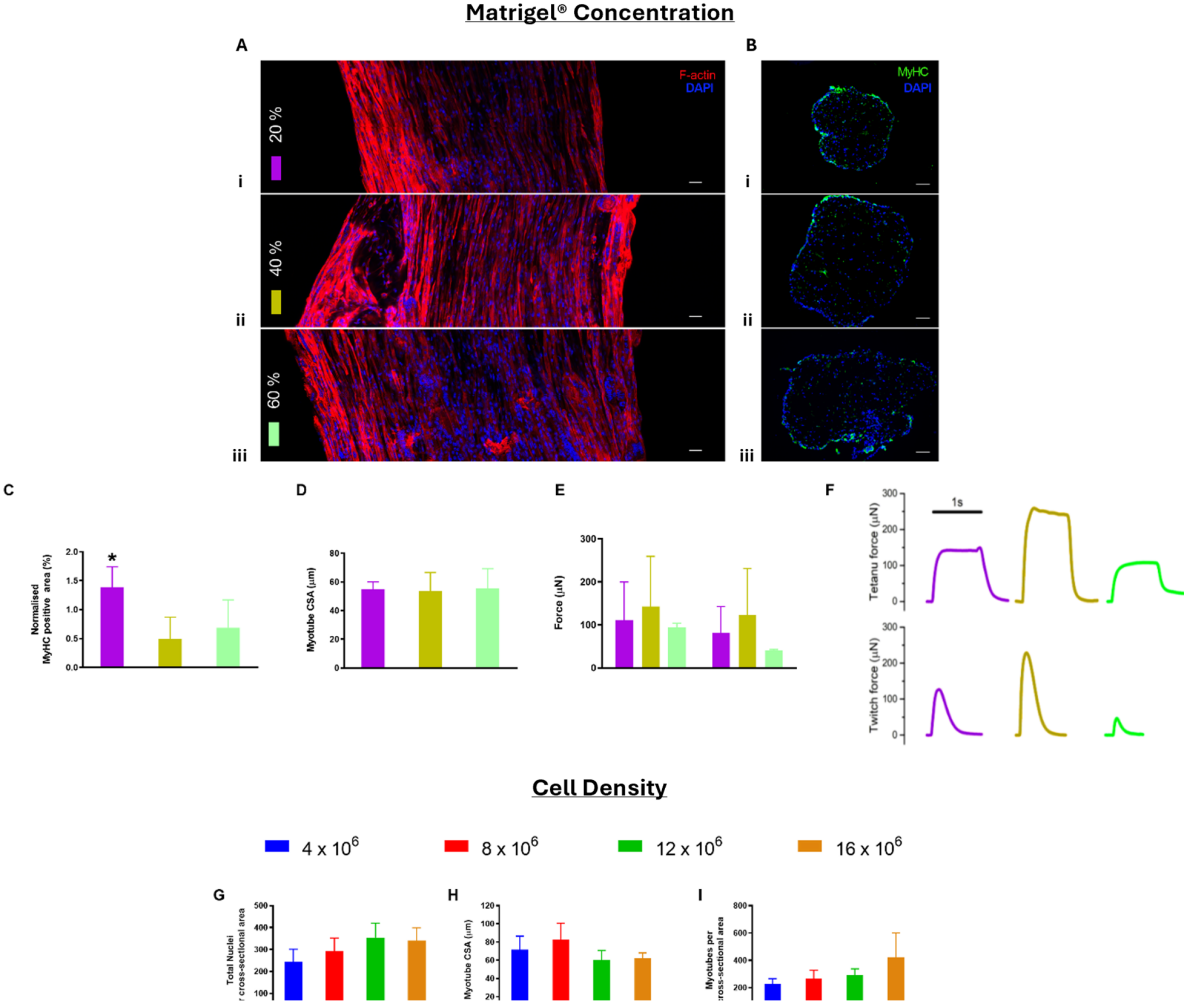
SM model composition. The impact of Matrigel^®^ concentration (a–f) and cell density (g–i) on SM parameters: (a) longitudinal images evidence the presence of mature SM structures in constructs composed of (i) 20%, (ii) 40% and (iii) 60 Matrigel^®^ (red: F-actin, blue: DAPI, scale bars = 100 µm), (b) cross sections displaying MyHC (green) and DAPI (blue) at all Matrigel^®^ concentrations (scale bars = 50 µm), (c) normalised MyHC positive area (%) from quantification of fluorescence staining, (d) myotube CSA (µm), (e) quantitative force (µN) measurements for tetanus and twitch, (f) graphical representation of individual tetanus and twitch measurements, (g) quantification of total nuclei per CSA for all cell concentrations, (h) quantification of myotube CSA (µm), and (i) representation of normalised MyHC positive area (%) after fluorescence quantification. (*N* = 3).

Immunofluorescent detection of longitudinal F-actin structures (Figure 2(a)) and MyHC (Figure 2(b)) confirmed the presence of mature myotubes within longitudinal and cross-sectional images for all SM models, irrespective of Matrigel^®^ concentration (Figure 2(a) and (b), i–iii). Increasing concentration of Matrigel^®^ beyond 20% within models led to a reduction in normalised MyHC expression, significantly reducing expression from 1.39% ± 0.35% (20% Matrigel^®^) to 0.49% ± 0.38% (*p < 0.05, 40% Matrigel^®^) and 0.69% ± 0.48% (*p < 0.05, 60% Matrigel^®^; Figure 2(c)). Average myotube cross sectional area (CSA) remained consistent irrespective of Matrigel^®^ concentration (Figure 2(d)). No significant differences were observed in peak tetanus or peak twitch force production (Figure 2(e) and (f)).

We next evaluated the impact of cell seeding density on skeletal muscle models containing 20% Matrigel^®^. C2C12 cells were cultured at increasing concentrations of 4, 8, 12 and 16 × 10^6^ cells/mL. Total nuclei per CSA (Figure 2(g)), myotube CSA (Figure 2(h)) and the number of myotubes per CSA were not found to be statistically different for any of the cell densities analysed (Figure 2(i)).

### Extracellular vesicle dynamics in 3D SM models

SM constructs were engineered using 20% Matrigel^®^ and a seeding density of 12 × 10^6^ cells/mL. These parameters maximised throughput and minimised cost/resources and provided a significant increase in MyHC expression, the most abundant native marker in mature SM (Figure 2(b) and (c)).

**Figure 3.**
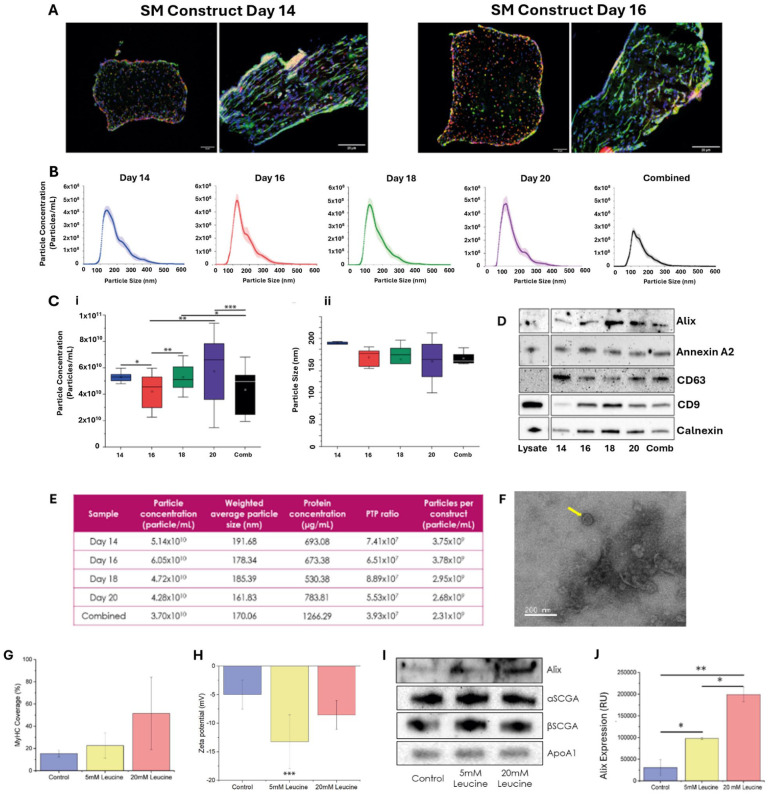
Profiling EVs dynamics in an SM model: (a) cross sectional and longitudinal immunofluorescence images of 3D SM model at days 14 and 16 of differentiation (green: MyHC, red: Rhodamine and blue: DAPI). Scale bar: 20 µm, (b) particle distribution for samples collected at day 14, day 16, day 18, day 20 and combined samples, (c) boxplots displaying (i) average particle concentration measurements and (ii) average particle sizes, (d) western blots displaying Alix, Annexin A2, CD63, CD9 and calnexin at days 14, 16, 18, 20 and combined (comb), (e) table presenting particle concentration, average size, protein concentration, particle-to-protein (PTP) ratio and concentration of particles isolated per individual construct, (f) representative TEM image displaying EV material isolated using the UF + SEC protocol, (g) percentage MyHC coverage, (h) zeta potential following supplementation with leucine, (i) western blot for Alix, α-SCGA, β-SCGA and APOA1, and (j) semi-quantitation of Alix expression in leucine supplemented SM model (*N* = 3). *p < 0.05. **p < 0.01. ***p > 0.001.

EVs were isolated from mature SM constructs (Figure 3(a)) using an optimised SEC + UF protocol.^39^ Particle size distributions ranged from 60 to 400 nm. Size distributions between days 14 to day 20 were similar, with mode sizes of 136.5 (day 14), 126.5 nm (day 16), 124.5 nm (day 18 and 20; Figure 3(b)). Variations in particle concentration were observed between day 14 and day 16 (*p < 0.05), day 16 and day 18 (**p < 0.01), day 16 and day 20 (**p < 0.01), day 18 and combined samples (*p < 0.05) and day 20 and combined samples (***p < 0.001; Figure 3(c)i). While the size range of particles varied, average particle sizes were over 160 nm at all timepoints analysed (Figure 3(c)ii). Overlapping interquartile ranges indicated minimal variation in particle size, supporting a stable and homogeneous population throughout differentiation, where no significant differences were observed. EV protein markers Alix, Annexin A2, CD63 and CD9 were identified at all timepoints analysed. . The ER marker calnexin was also identified at all timepoints (Figure 3(d) and Supplemental Figure 1). No significant variations were observed in protein concentration (*p* > 0.05) or particle-to-protein (PTP) ratios, the latter often applied as a rudimentary indicator of sample purity (Figure 3(e)). Combined fractions had a wider size distribution with the highest protein concentration and lowest PTP ratio (Figure 3(e)). TEM imaging confirmed the presence of characteristic EV material (Figure 3(f)).

We next analysed the effect of leucine supplementation on EV yield and composition at day 14, based on its previously described effects on muscle hypertrophy.^40^ MyHC coverage increased following the addition of leucine. However, this trend was not significant (Figure 3(g)). The zeta potential of particles was found to decrease significantly (**p < 0.01) following 5 mM leucine supplementation. While the addition of 20 mM did not induce a change in zeta potential relative to the untreated control (Figure 3(h)). Finally, we reported the presence of a marker of EV biogenesis (Alix) and markers of the sarcoplasmic reticulum (α-sarcoglycan and β-sarcoglycan, α- and β-SCGA) in the EV fraction (Figure 3(i) and Supplemental Figure 3). APOA1 was identified in all fractions. Alix expression significantly increased following leucine dosing, with increased expression observed for the 20 mM leucine group (*p < 0.05, **p < 0.01). SM markers α- and β-SCGA did not vary in intensity following leucine treatment (*p* > 0.05; Figure 3(i) and (j)).

## Discussion

We developed a 3D SM model and provided a demonstration of its application for the study of EV dynamics. Tissue engineering SM models have the potential to provide a valuable intermediate platform to bridge 2D cell culture studies and complex in vivo experiments. These models provide advantages over comparatively complex in vivo systems when attempting to study poorly defined processes such as intra- and inter-cellular communication events, which can become increasingly challenging in the presence of multiple cell types. This is true for studies in which tracing the precise cellular origin of EVs found in biological fluids such as blood plasma still presents a major challenge due to the absence of defined tissue-specific markers. While several bioengineered 3D models exist for the study of SM,^41–43^ to the best of our knowledge, none has been applied to the study of SM-EVs. Given that bioengineered 3D SM models have previously been shown to better mirror native muscle morphology and contractile performance, the optimisation of an SM model for the study of EV dynamics is of value to the field.^44,45^ Previous 2D studies have shown that SM-EV production and content can be modulated by the application of a mechanical or electrical stimuli. For example, increased EV secretion was described following the mechanical stimulation of human primary muscle cells (HPMCs) grown on fibrinogen scaffolds through the activation of yes-associated protein (YAP).^46^ While the induction of chronic contractile activity in C2C12 myotubes by electrical stimulation was found to increase the production of small EVs, which induced exercise-associated changes such as mitochondrial biogenesis and increased ATP production when exogenously administered to recipient C2C12 myoblasts in vitro.^47^ Equally important to the study of SM-EVs is the specificity of the EV isolation protocol applied. In many of these previous studies, EVs were isolated by differential ultracentrifugation (dUC) or using commercial precipitation methods (ExoQuick-TC and Total Exosome Isolation Reagent). It has been widely demonstrated that the application of dUC - and particularly commercial precipitation kits - can yield heterogeneous and low purity EV preparations.^48–50^ Both protocols typically result in the inclusion of protein aggregates and/or lipoproteins of similar size and density.^51,52^ As such, the present study applied an optimised SEC + UF isolation method previously developed by our group to reduce the inclusion of NVEPs .^39^ While this simple and low-cost method has been shown to offer enhanced levels of purity, it should be noted that it does not remove the presence of all NVEPs from EV preparations, with some APOA1 identified in our preparations (Figure 3(i)). Additional processing steps (e.g. additional filtration or ion exchange chromatography) could be applied to further increase sample purity. However, these steps will add additional processing time and costs.^53^ A similar SEC + UF protocol has since been applied for the recovery of SM-EVs from C2C12 cells incorporated on a fibrin gel containing 20% Matrigel^®54^. In the study by Murata et al. (2023) electrically stimulated their cell culture to generate a hypertrophic stimulus. Notably, the EVs isolated displayed a significant increase in Alix expression following tetanic electrical stimulation at 30 Hz. This trend was reflective of qualitative observations from the present study that documented an increase in Alix^+^ SM-EVs following leucine supplementation (Figure 3). There is some evidence to suggest that EV secretion and content can be modulated by nutritional changes and amino acid supplementation.^55^ While Alix has previously been identified in EV preparations obtained from both C2C12 myoblasts and myotubes.^10,56^ In addition to its role in EV biogenesis, within SM Alix also functions as a substrate for a ubiquitin ligase protein that is closely related to the dynamics of actin and the status of myotubes.^57^ These ubiquitin ligase-Alix complexes cooperate in maintaining SM structure and functionality through mitochondrial regulation.^57-59^ Hence, there is a requirement to better understand the diverse functions of this protein during myogenesis and further studies are required to determine that variations in Alix expression are specific to EVs and not reflective of broader changes in the cell.^13^

In addition to markers of EV biogenesis, we analysed the expression of prospective muscle-specific markers (α-SCGA and β-SCGA) to validate their presence on SM-EVs in a defined SM culture system. SCGA proteins are crucial in SM contraction and Ca^2+^ metabolism.^60,61^ The presence of α-SCGA has previously been reported in SM-EV samples from mouse serum using polyethylene glycol (PEG) isolation^62^ and from rat and human plasma using density gradient UC with^38^ and without immunoaffinity capture.^63^ Both isoforms could be identified within our SM-EV fractions (Figure 3), validating previous findings, and highlighting high levels of expression of sarcoglycans in SM-EV preparations. Notably, leucine supplementation did not appear to modulate the expression of α- or β-SCGA in SM-EVs. A study by Guescini et al. (2015) proposed α-SCGA as a potential marker of SM-derived EVs in plasma.^38^ However, subsequent studies failed to identify α-SCGA ^+^ EVs before and after exercise training.^64^ Findings from the present study suggest that SR components (SCGA and calnexin) are recovered in SM-EV preparations. Importantly, these findings perhaps further support a role for the SR/ER in regulating EV secretion.^65^ However, additional studies are required in which SCGA expression is quantified across a range of EV preparations to fully establish its potential as an SM-EV marker. Comparisons should specifically focus on tissues where α-SCGA and β-SCGA expression has been recorded. This includes cardiac muscle, the diaphragm and central nervous system.^66^ In addition to SCGAs, our data demonstrated that the ER localised protein calnexin could be detected in all fractions analysed. Calnexin is commonly regarded as a negative marker indicative of ER contamination. Notably, we did not observe the presence of this marker in previous 2D studies with C2C12 or primary human skeletal muscle cells.^39,67^ Its expression in the present study may be attributable to increased cell stress resulting from 3D culture, which requires further investigation. However, it is also notable that EVs recovered from 3D models were comparatively large with weighted average particle sizes of >160 nm recorded for all time points analysed. While the presence of calnexin is not ubiquitous throughout SM-EV studies, its presence has been reported in large EVs (e.g. EVs isolated using lower g-force UC protocols or from earlier SEC fractions, such as fractions 5 and 6) from SM and other cell types.^68,69^ As such, it is possible that the presence of calnexin in the present study could reflect the inclusion of larger EVs likely recovered in earlier SEC fractions. While there have been some reports of differential protein and miRNA content in small and large myotube EVs and their respective responses to electrical pulse stimulation, we identify a need for further investigation into their respective functions in SM physiology.^70^ Our findings provide important complementary data to indicate that protein associated with the sarcoplasmic/endoplasmic reticulum are present in SM-EV preparations. We highlight the need for additional studies to confirm whether the expression of these markers and to confirm their association with small or larger SM-EVs in vitro and in vivo.

In this study we documented the development and application of a bioengineered SM model for the study of EVs. SM-EVs expressed multiple markers of EV biogenesis and were positive for the presence of SGCAs, supporting their potential as prospective future biomarkers for in vivo studies but identifying the need for future direct comparisons with EVs isolated from a range of other tissue sources. Despite the application of a previously optimised EV isolation method, the ER localised protein calnexin was consistently detected in all EV fractions. Its expression supports previous studies demonstrating the presence of calnexin in larger EV populations but could also be indicative of cellular stress encountered in prolonged 3D culture. As such, it requires further investigation. Finally, we emphasise potential future applications of our SM model as a biomimetic platform to further investigate the effects of physical stimulation on SM-EV yield and content or in the in vitro study of SM crosstalk with organs such as bone, adipose tissue and the nervous system.^71,72^

## Supplemental Material

sj-docx-1-tej-10.1177_20417314261427541 – Supplemental material for Engineering a skeletal muscle model to study extracellular vesicle dynamics Supplemental material, sj-docx-1-tej-10.1177_20417314261427541 for Engineering a skeletal muscle model to study extracellular vesicle dynamics by María Fernández-Rhodes, Rowan Rimington, Jacob Fleming, Andrew J. Capel, Owen G. Davies and Mark P. Lewis in Journal of Tissue Engineering

## References

[1] Baldwin MJ Cribbs AP Guilak F , et al. Mapping the musculoskeletal system one cell at a time. Nat Rev Rheumatol 2021; 17(5): 247–248. 33712803 10.1038/s41584-021-00600-7

[2] Frontera WR Ochala J. Skeletal muscle: a brief review of structure and function. Behav Genet 2015; 45(2): 183–195. 10.1007/s00223-014-9915-y 25294644

[3] Lowe DA Alway SE. Animal models for inducing muscle hypertrophy: are they relevant for clinical applications in humans? J Orthop Sports Phys Ther 2002; 32(2): 36–43. 11838579 10.2519/jospt.2002.32.2.36

[4] Dessauge F Schleder C Perruchot MH , et al. 3D in vitro models of skeletal muscle: myopshere, myobundle and bioprinted muscle construct. Vet Res.BioMed Central 2021; 52(1): 1–12. 10.1186/s13567-021-00942-w PMC8136231 34011392

[5] Wang J Khodabukus A Rao L , et al. Engineered skeletal muscles for disease modeling and drug discovery. Biomaterials 2019; 221: 119416. 31419653 10.1016/j.biomaterials.2019.119416 PMC7041662

[6] Joshi S Lee WH Chen P , et al. Editorial: 3D cell culture systems for cardiovascular tissue engineering: in vitro modelling and in vivo regenerative therapies. Front Cardiovasc Med 2021; 8: 675676. 34222371 10.3389/fcvm.2021.675676 PMC8247440

[7] Gomarasca M Banfi G Lombardi G (eds). Myokines: the endocrine coupling of skeletal muscle and bone. 1st ed. Elsevier Inc, 2020. Vol.94. 10.1016/bs.acc.2019.07.010 31952571

[8] Tagliaferri C Wittrant Y Davicco MJ , et al. Muscle and bone, two interconnected tissues. Ageing Res Rev 2015; 21: 55–70. 25804855 10.1016/j.arr.2015.03.002

[9] Severinsen MCK Pedersen BK . Muscle-organ crosstalk: the emerging roles of myokines. Endocr Rev 2020; 41(4): 594–609. 32393961 10.1210/endrev/bnaa016 PMC7288608

[10] Forterre A Jalabert A Berger E , et al. Proteomic analysis of C2C12 myoblast and myotube exosome-like vesicles: a new paradigm for myoblast-myotube cross talk? PLoS One 2014; 9(1): e84153. 10.1371/journal.pone.0084153 PMC3879278 24392111

[11] Suire CN Eitan E Shaffer NC , et al. Walking speed decline in older adults is associated with elevated pro-BDNF in plasma extracellular vesicles. Exp Gerontol 2017; 98: 209–216. 28843509 10.1016/j.exger.2017.08.024 PMC5642035

[12] Trovato E Di Felice V Barone R. Extracellular vesicles: delivery vehicles of myokines. Front Physiol 2019; 10: 522. 31133872 10.3389/fphys.2019.00522 PMC6514434

[13] Rome S Forterre A Mizgier ML , et al. Skeletal muscle-released extracellular vesicles: state of the art. Front Physiol 2019; 10: 929. 31447684 10.3389/fphys.2019.00929 PMC6695556

[14] Le Bihan MC Bigot A Jensen SS , et al. In-depth analysis of the secretome identifies three major independent secretory pathways in differentiating human myoblasts. J Proteomics 2012; 77: 344–356. 23000592 10.1016/j.jprot.2012.09.008

[15] Baci D Chirivì M Pace V , et al. Extracellular vesicles from skeletal muscle cells efficiently promote myogenesis in induced pluripotent stem cells. Cells 2020; 9(6): 1–23. 10.3390/cells9061527 PMC7349204 32585911

[16] Guescini M Maggio S Ceccaroli P , et al. Extracellular vesicles released by oxidatively injured or intact C2C12 myotubes promote distinct responses converging toward myogenesis. Int J Mol Sci 2017; 18(11): 2488. 10.3390/ijms18112488 PMC5713454 29165341

[17] Aswad H Jalabert A Rome S. Depleting extracellular vesicles from fetal bovine serum alters proliferation and differentiation of skeletal muscle cells in vitro. BMC Biotechnol 2016; 16(1): 32. 27038912 10.1186/s12896-016-0262-0 PMC4818850

[18] Rome S. Muscle and adipose tissue communicate with extracellular vesicles. Int J Mol Sci 2022; 23(13): 7052. 35806052 10.3390/ijms23137052 PMC9266961

[19] Jalabert A Reininger L Berger E , et al. Profiling of ob/ob mice skeletal muscle exosome-like vesicles demonstrates combined action of miRNAs, proteins and lipids to modulate lipid homeostasis in recipient cells. Sci Rep 2021; 11(1): 21626. 34732797 10.1038/s41598-021-00983-3 PMC8566600

[20] Lara-Castillo N Johnson ML. Bone-muscle mutual interactions. Curr Osteoporos Rep 2020; 18(4): 408–421. 32519283 10.1007/s11914-020-00602-6 PMC8059358

[21] Maurel DB Jähn K Lara-Castillo N. Muscle-bone crosstalk: emerging opportunities for novel therapeutic approaches to treat musculoskeletal pathologies. Biomedicines 2017; 5(4): 62. 29064421 10.3390/biomedicines5040062 PMC5744086

[22] Lamichhane TN Sokic S Schardt JS , et al. Emerging roles for extracellular vesicles in tissue engineering and regenerative medicine. Tissue Eng Part B: Rev 2015; 21(1): 45–54. 24957510 10.1089/ten.teb.2014.0300 PMC4321981

[23] Conkright WR Beckner ME Sterczala AJ , et al. Resistance exercise differentially alters extracellular vesicle size and subpopulation characteristics in healthy men and women: an observational cohort study. Physiol Genomics 2022; 54(9): 350–359. 35816651 10.1152/physiolgenomics.00171.2021

[24] Frühbeis C Helmig S Tug S , et al. Physical exercise induces rapid release of small extracellular vesicles into the circulation. J Extracell Vesicles 2015; 4(1): 1–11. 10.3402/jev.v4.28239 PMC4491306 26142461

[25] Garner RT Solfest JS Nie Y , et al. Multivesicular body and exosome pathway responses to acute exercise. Exp Physiol 2020; 105(3): 511–521. 31917487 10.1113/EP088017

[26] Khodabukus A Prabhu N Wang J , et al. In vitro tissue-engineered skeletal muscle models for studying muscle physiology and disease. Adv Healthc Mater 2018; 7(15): e1701498. 10.1002/adhm.201701498 PMC6105407 29696831

[27] Jalal S Dastidar S Tedesco FS. Advanced models of human skeletal muscle differentiation, development and disease: three-dimensional cultures, organoids and beyond. Curr Opin Cell Biol 2021; 73: 92–104. 34384976 10.1016/j.ceb.2021.06.004 PMC8692266

[28] Capel AJ Rimington RP Fleming JW , et al. Scalable 3D printed molds for human tissue engineered skeletal muscle. Front Bioeng Biotechnol 2019; 7: 1–13. 30838203 10.3389/fbioe.2019.00020 PMC6383409

[29] Wragg NM Mosqueira D Blokpeol-Ferreras L , et al. Development of a 3D tissue-engineered skeletal muscle and bone co-culture system. Biotechnol J 2020; 15(1): e1900106. 10.1002/biot.201900106 31468704

[30] Rimington RP Capel AJ Chaplin KF , et al. Differentiation of bioengineered skeletal muscle within a 3D printed perfusion bioreactor reduces atrophic and inflammatory gene expression. ACS Biomater Sci Eng 2019; 5(10): 5525–5538. 33464072 10.1021/acsbiomaterials.9b00975

[31] Vandenburgh H. High-content drug screening with engineered musculoskeletal tissues. Tissue Eng Part B: Rev 2010; 16(1): 55–64. 19728786 10.1089/ten.teb.2009.0445 PMC2865990

[32] Fleming JW Capel AJ Rimington RP , et al. Bioengineered human skeletal muscle capable of functional regeneration. BMC Biol 2020; 18(1): 145. 33081771 10.1186/s12915-020-00884-3 PMC7576716

[33] Fleming JW Capel AJ Rimington RP , et al. Functional regeneration of tissue engineered skeletal muscle in vitro is dependent on the inclusion of basement membrane proteins. Cytoskeleton 2019; 76(6): 371–382. 31376315 10.1002/cm.21553 PMC6790946

[34] Aguilar-Agon KW Capel AJ Fleming JW , et al. Mechanical loading of tissue engineered skeletal muscle prevents dexamethasone induced myotube atrophy. J Muscle Res Cell Motil 2021; 42(2): 149–159. 32955689 10.1007/s10974-020-09589-0 PMC8332579

[35] Khodabukus A Madden L Prabhu NK , et al. Electrical stimulation increases hypertrophy and metabolic flux in tissue-engineered human skeletal muscle. Biomaterials 2019; 198: 259–269. 30180985 10.1016/j.biomaterials.2018.08.058 PMC6395553

[36] Woo HK Cho YK Lee CY , et al. Characterization and modulation of surface charges to enhance extracellular vesicle isolation in plasma. Theranostics 2022; 12(5): 1988–1998. 35265194 10.7150/thno.69094 PMC8899565

[37] Caulfield MP Li S Lee G , et al. Direct determination of lipoprotein particle sizes and concentrations by ion mobility analysis. Clin Chem 2008; 54(8): 1307–1316. 18515257 10.1373/clinchem.2007.100586

[38] Guescini M Canonico B Lucertini F , et al. Muscle releases alpha-sarcoglycan positive extracellular vesicles carrying miRNAs in the bloodstream. PLoS One 2015; 10(5): e0125094. 10.1371/journal.pone.0125094 PMC4425492 25955720

[39] Fernández-Rhodes M Adlou B Williams S , et al. Defining the influence of size-exclusion chromatography fraction window and ultrafiltration column choice on extracellular vesicle recovery in a skeletal muscle model. J Extracell Biol 2023; 2(4): e85. 10.1002/jex2.85 PMC11080914 38939692

[40] Martin NRW Turner MC Farrington R , et al. Leucine elicits myotube hypertrophy and enhances maximal contractile force in tissue engineered skeletal muscle in vitro. J Cell Physiol 2017; 232(10): 2788–2797. 28409828 10.1002/jcp.25960 PMC5518187

[41] Pinton L Khedr M Lionello VM , et al. 3D human induced pluripotent stem cell-derived bioengineered skeletal muscles for tissue, disease and therapy modeling. Nat Protoc 2023; 18(4): 1337–1376. 36792780 10.1038/s41596-022-00790-8

[42] Fleming JW Capel AJ Rimingtion RP , et al. Bioengineered human skeletal muscle with a Pax7+ satellite cell niche capable of functional regeneration. bioRxiv 2020. doi:10.1101/2020.05.04.076828 PMC7576716 33081771

[43] Wang K Smith SH Iijima H , et al. Bioengineered 3D skeletal muscle model reveals complement 4b as a cell-autonomous mechanism of impaired regeneration with aging. Adv Mater 2023; 35(17): e2207443. 10.1002/adma.202207443 PMC13175247 36650030

[44] Gao L Li L Wu W , et al. 3D bio-printed in vitro skeletal muscle with pennate fibers architecture to enhance contractile function. Int J Bioprinting 2024; 0(0): 4371.

[45] Smith AS Luttrell SM Dupont JB , et al. High-throughput, real-time monitoring of engineered skeletal muscle function using magnetic sensing. J Tissue Eng 2022; 13: 20417314221122127. 10.1177/20417314221122127 PMC9445471 36082311

[46] Guo S Debbi L Zohar B , et al. Stimulating extracellular vesicles production from engineered tissues by mechanical forces. Nano Lett 2021; 21(6): 2497–2504. 33709717 10.1021/acs.nanolett.0c04834

[47] Obi PO Souza TFG Özerkliğ B , et al. Extracellular vesicles released from skeletal muscle post-chronic contractile activity increase mitochondrial biogenesis in recipient myoblasts. J Extracell Vesicles 2025; 14(4): e70045. 10.1002/jev2.70045 PMC11982704 40205946

[48] Coumans FAW Brisson AR Buzas EI , et al. Methodological guidelines to study extracellular vesicles. Circ Res 2017; 120(10): 1632–1648. 28495994 10.1161/CIRCRESAHA.117.309417

[49] Sidhom K Obi PO Saleem A. A review of exosomal isolation methods: is size exclusion chromatography the best option? Int J Mol Sci 2020; 21(18): 1–19. 10.3390/ijms21186466 PMC7556044 32899828

[50] Williams S Fernandez-Rhodes M Law A , et al. Comparison of extracellular vesicle isolation processes for therapeutic applications. J Tissue Eng 2023; 14: 204173142311746. 10.1177/20417314231174609 PMC10214056 37251735

[51] Sódar BW Kittel Á Pálóczi K , et al. Low-density lipoprotein mimics blood plasma-derived exosomes and microvesicles during isolation and detection. Sci Rep 2016; 6(1): 12. 27087061 10.1038/srep24316 PMC4834552

[52] Issman L Brenner B Talmon Y , et al. Cryogenic transmission electron microscopy nanostructural study of shed microparticles. PLoS One 2013; 8(12): e83680. 10.1371/journal.pone.0083680 PMC3873325 24386253

[53] Wang Y Zhang Y Li Z , et al. Combination of size-exclusion chromatography and ion exchange adsorption for improving the proteomic analysis of plasma-derived extracellular vesicles. Proteomics 2023; 23(9): e2200364. 10.1002/pmic.202200364 36624553

[54] Fan SJ Kroeger B Marie PP , et al. Glutamine deprivation alters the origin and function of cancer cell exosomes. EMBO J 2020; 39(16): e103009. 10.15252/embj.2019103009 PMC7429491 32720716

[55] Guescini M Guidolin D Vallorani L , et al. C2C12 myoblasts release micro-vesicles containing mtDNA and proteins involved in signal transduction. Exp Cell Res 2010; 316(12): 1977–1984. 20399774 10.1016/j.yexcr.2010.04.006

[56] Bongiovanni A Romancino DP Campos Y , et al. Alix protein is substrate of ozz-E3 ligase and modulates actin remodeling in skeletal muscle. J Biol Chem 2012; 287(15): 12159–12171. 22334701 10.1074/jbc.M111.297036 PMC3320967

[57] Campos Y Rodriguez-Enriquez R Palacios G , et al. Mitochondrial proteostasis mediated by CRL5 Ozz and Alix maintains skeletal muscle function. bioRxiv 2023. doi:10.1101/2023.07.11.548601

[58] Romancino DP Paterniti G Campos Y , et al. Identification and characterization of the nano-sized vesicles released by muscle cells. FEBS Lett 2013; 587(9): 1379–1384. 23523921 10.1016/j.febslet.2013.03.012 PMC4714929

[59] Liu LA Engvall E. Sarcoglycan isoforms in skeletal muscle. J Biol Chem 1999; 274(53): 38171–38176. 10608889 10.1074/jbc.274.53.38171

[60] Berger J Berger J. The sarcoglycan complex in skeletal muscle. Front Biosci 2016; 21(4): 744–756. 10.2741/4418 26709803

[61] Fulzele S Mendhe B Khayrullin A , et al. Muscle-derived miR-34a increases with age in circulating extracellular vesicles and induces senescence of bone marrow stem cells. Aging 2019; 11(6): 1791–1803. 30910993 10.18632/aging.101874 PMC6461183

[62] Ismaeel A Van Pelt DW Hettinger ZR , et al. Extracellular vesicle distribution and localization in skeletal muscle at rest and following disuse atrophy. Skelet Muscle 2023; 13(1): 6. 36895061 10.1186/s13395-023-00315-1 PMC9999658

[63] Brahmer A Neuberger E Esch-Heisser L , et al. Platelets, endothelial cells and leukocytes contribute to the exercise-triggered release of extracellular vesicles into the circulation. J Extracell Vesicles 2019; 8(1): 1615820. 10.1080/20013078.2019.1615820 PMC6542154 31191831

[64] Wenzel EM Raiborg C. ER-endosome contacts master the ins and outs of secretory endosomes. J Cell Biol 2022; 221(12): 10–12. 10.1083/jcb.202210033 PMC9652769 36355088

[65] Nicita F Freni J Centofanti A , et al. A scoping review of sarcoglycan expression in non-muscle organs: beyond muscles. Biomolecules 2025; 15(7): 1020. 10.3390/biom15071020 PMC12294094 40723892

[66] Fernández-Rhodes M Buchan E Gagnon SD , et al. Extracellular vesicles may provide an alternative detoxification pathway during skeletal muscle myoblast ageing. J Extracell Biol 2024; 3(8): e171. 10.1002/jex2.171 PMC11336379 39169919

[67] Saludas L Garbayo E Ruiz-Villalba A , et al. Isolation methods of large and small extracellular vesicles derived from cardiovascular progenitors: a comparative study. Eur J Pharm Biopharm 2022; 170: 187–196. 34968647 10.1016/j.ejpb.2021.12.012

[68] Crescitelli R Lässer C Jang SC , et al. Subpopulations of extracellular vesicles from human metastatic melanoma tissue identified by quantitative proteomics after optimized isolation. J Extracell Vesicles 2020; 9(1): 1722433. 32128073 10.1080/20013078.2020.1722433 PMC7034452

[69] Aas V Øvstebø R Brusletto BS , et al. Distinct microRNA and protein profiles of extracellular vesicles secreted from myotubes from morbidly obese donors with type 2 diabetes in response to electrical pulse stimulation. Front Physiol 2023; 14: 1143966. 37064893 10.3389/fphys.2023.1143966 PMC10098097

[70] Wu Z Gao Y Chen Q , et al. Inter-organ crosstalk and regulatory mechanisms of skeletal muscle-derived extracellular vesicles in systemic metabolic homeostasis. J Cell Mol Med 2025; 29(21): e70896. 10.1111/jcmm.70896 PMC12587308 41190649

[71] Jia J Wang L Zhou Y , et al. Muscle-derived extracellular vesicles mediate crosstalk between skeletal muscle and other organs. Front Physiol 2024; 15: 1501957. 39844898 10.3389/fphys.2024.1501957 PMC11750798

[72] Murata A Akiyama H Honda H , et al. Electrical pulse stimulation-induced tetanic exercise simulation increases the secretion of extracellular vesicles from C2C12 myotubes. Biochem Biophys Res Commun 2023; 672: 177–184. 37354611 10.1016/j.bbrc.2023.06.054

