# Supplementary material for: Engineering a skeletal muscle model to study extracellular vesicle dynamics
Source: J Tissue Eng. 2026 Apr 5;17:20417314261427541. doi: 10.1177/20417314261427541 (PMC13053972; doi:10.1177/20417314261427541)

**Supplementary Table 1:** Western Blot antibody guide for targeted proteins. We included information about dilutions, source, supplier and codes and secondary antibody dilutions used in the results represented in this study.

| Primary Antibody | Source | Dilution | Supplier | Secondary antibody | Dilution |
| --- | --- | --- | --- | --- | --- |
| Anti-Alix | Rabbit | 1:1000 | Santa Cruz (sc-53540) | Anti-Rabbit | 1:3000 |
| Anti-Annexin A2 | Rabbit | 1:1000 | Abcam (ab41803) | Anti-Rabbit | 1:3000 |
| Anti- TSG101 | Rabbit | 1:1000 | Abcam (ab30871) | Anti-Rabbit | 1:3000 |
| Anti-CD9 | Rabbit | 1:1000 | Abcam (ab92726) | Anti-Rabbit | 1:2000 |
| Anti-CD63 | Rabbit | 1:1000 | Abcam (ab216130) | Anti-Rabbit | 1:3000 |
| Anti-Calnexin | Mouse | 1:1000 | Abcam (ab22595) | Anti-Mouse | 1:3000 |
| Anti-ApoA1 | Rabbit | 1:1000 | Abcam  (ab20453) | Anti-Rabbit | 1:3000 |
| Anti-α-sarcoglycan | Rabbit | 1:500 | Abcam (ab189254) | Anti-Rabbit | 1:3000 |
| Anti-β-sarcoglycan | Rabbit | 1:500 | Abcam (ab135954) | Anti-Rabbit | 1:3000 |

**Supplementary Figure 1:** Quantification of western blot data presented in Figure 3D.

**
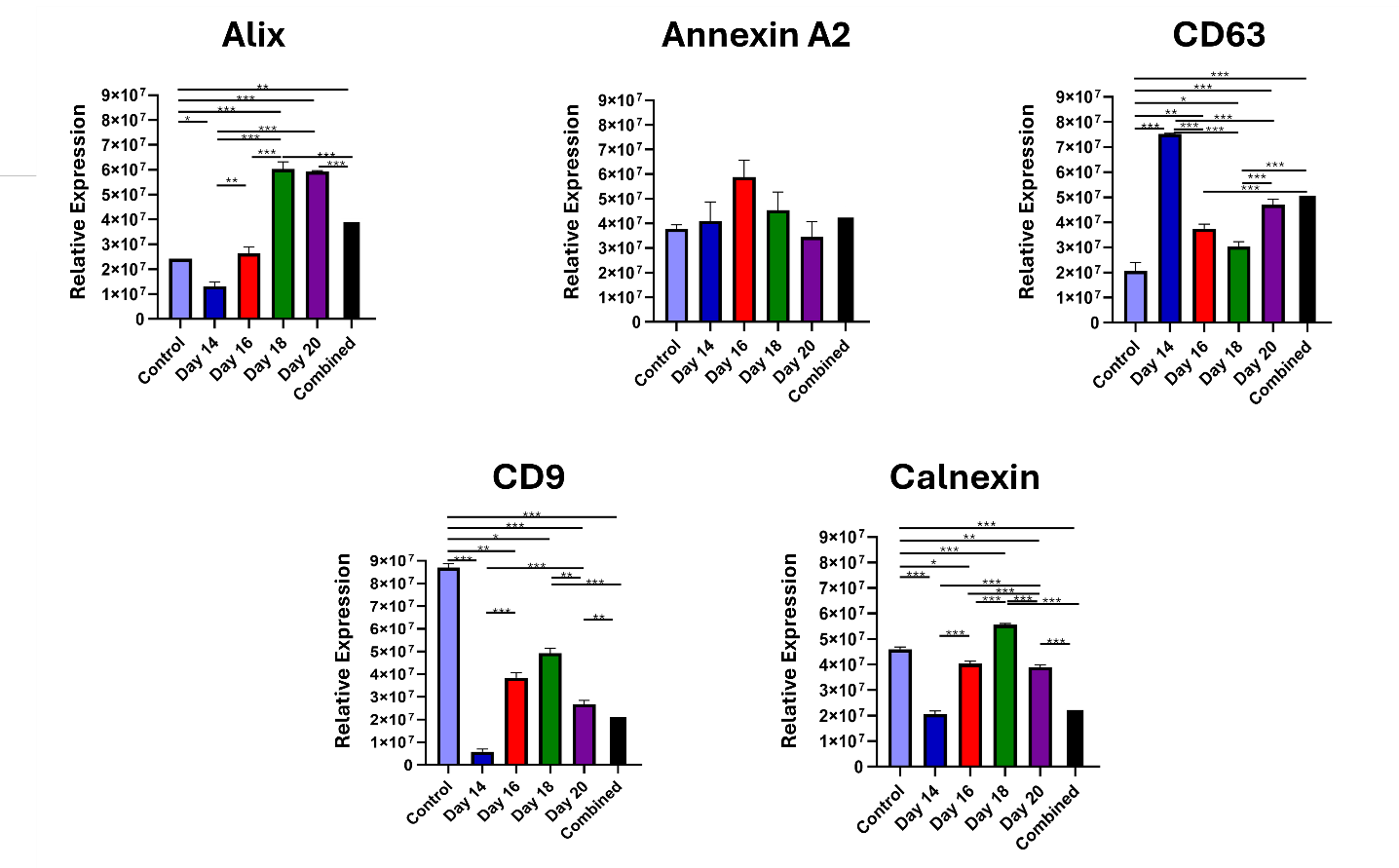
**

**Supplementary Figure 2:** Quantification of western blot data presented in Figure 3I.


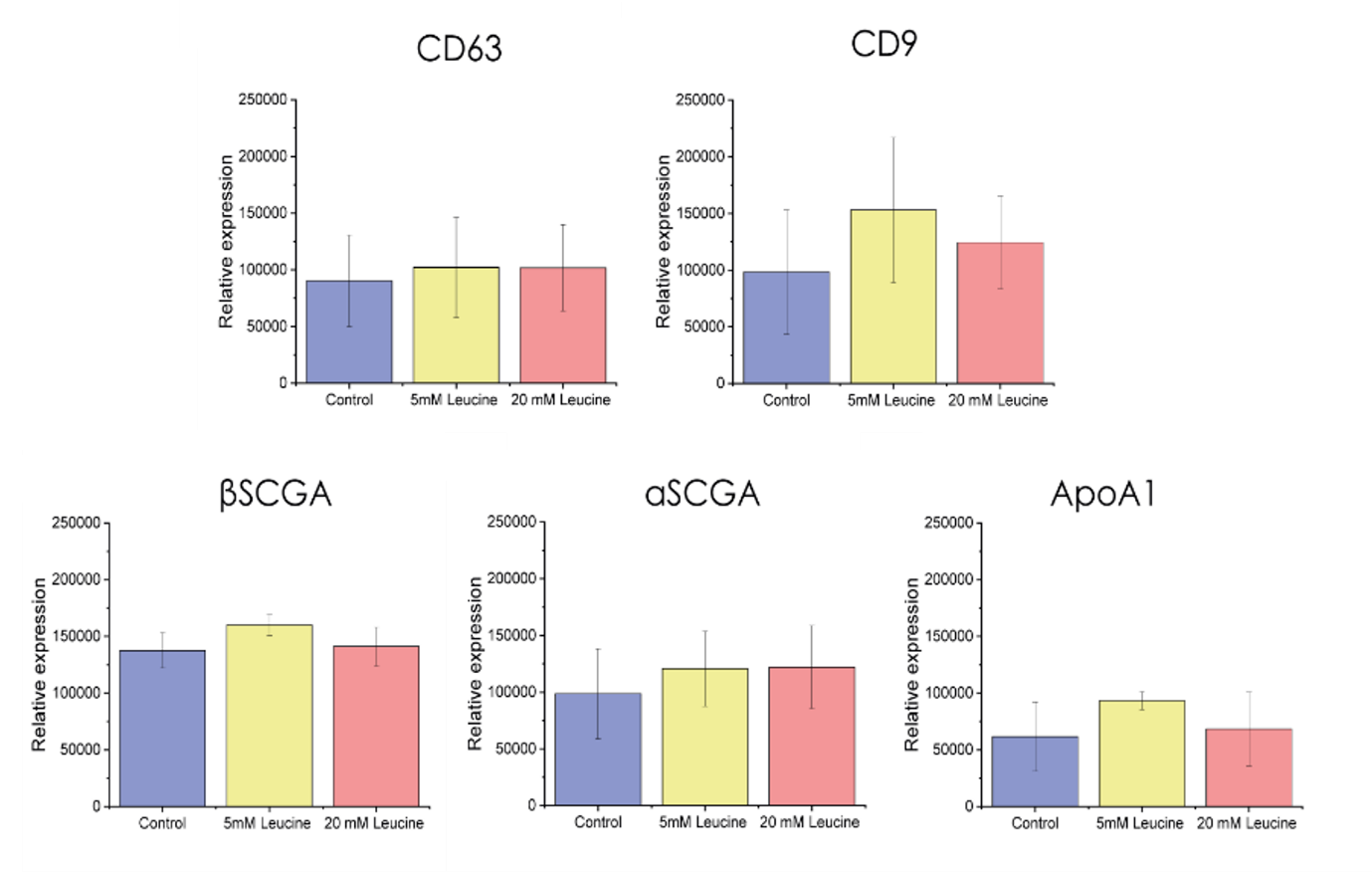

Supplement: sj-docx-1-tej-10.1177_20417314261427541 – Supplemental material for Engineering a skeletal muscle model to study extracellular vesicle dynamics [file sj-docx-1-tej-10.1177_20417314261427541.docx]
